# Cysteine-rich receptor-like kinase CRK5 as a regulator of growth, development, and ultraviolet radiation responses in *Arabidopsis thaliana*

**DOI:** 10.1093/jxb/erv143

**Published:** 2015-05-12

**Authors:** Paweł Burdiak, Anna Rusaczonek, Damian Witoń, Dawid Głów, Stanisław Karpiński

**Affiliations:** Department of Plant Genetics, Breeding and Biotechnology, Faculty of Horticulture, Biotechnology and Landscape Architecture, Warsaw University of Life Sciences - SGGW, Nowoursynowska 159, 02-776 Warsaw, Poland

**Keywords:** Cell death, cysteine-rich kinases (CRKs), ethylene (ET), reactive oxygen species (ROS), salicylic acid (SA), senescence.

## Abstract

In plants, receptor-like protein kinases play essential roles in signal transduction by recognizing extracellular stimuli and activating the downstream signalling pathways. Cysteine-rich receptor-like kinases (CRKs) constitute a large subfamily of receptor-like protein kinases, with 44 members in *Arabidopsis thaliana*. They are distinguished by the novel C-X8-C-X2-C motif (DUF26) in the extracellular domains. One of them, CRK5, is an important component of the biochemical machinery involved in the regulation of essential physiological processes. Functional characterization of *crk5* mutant plants showed their clear phenotype, manifested by impaired stomatal conductance and accelerated senescence. This phenotype correlated with accumulation of reactive oxygen species, higher foliar levels of ethylene and salicylic acid, and increased transcript abundance for genes associated with signalling pathways corresponding to these hormones. Moreover, the *crk5* plants displayed enhanced cell death and oxidative damage in response to ultraviolet radiation. Complementation of *CRK5* mutation managed to recover the wild-type phenotype, indicating an essential role of this gene in the regulation of growth, development, and acclimatory responses.

## Highlight

The functional characterization of *Arabidopsis thaliana crk5* mutant and complementation lines reveals an essential regulatory role of this gene in ROS homeostasis, which affects senescence and abiotic stress response.

## Introduction

A large number of receptor-like protein kinases (RLKs) evolved in plants as a response to a variety of external and internal stimuli. RLKs bridge the gap between the perception of these stimuli and the transmission of the input signal finally leading to the expression of appropriate target genes. They belong to a large gene family with more than 610 members, comprising nearly 2.5% of *Arabidopsis* protein-coding genes (Shiu and Bleecker, 2001). They are composed of an amino-terminal signal sequence, an extracellular domain, a single transmembrane domain, and a cytoplasmic domain with serine/threonine protein kinase activity (Czernic *et al.*, 1999). Microarray analysis showed that RLKs tend to be significantly over-represented among genes upregulated under biotic and abiotic stress conditions (especially UV-B, wounding, and osmotic stress) and presumably have a role in cell death regulation (Lehti-Shiu *et al.*, 2009). RLK family members are characterized by a great diversity of extracellular domains facilitating the perception of a wide range of signals, which the basis for their classification into subfamilies. A large subgroup of RLKs is constituted by cysteine-rich receptor-like kinases (CRKs), with 44 members in *Arabidopsis* (Wrzaczek *et al.*, 2010). They contain two copies of the conserved C-X8-C-X2-C motif (DUF26) in their extracellular region (Chen, 2001). The precise biochemical role of the DUF26 domain is still unclear but the cysteines have been suggested to form disulfide bridges as potential targets for thiol redox regulation and, thus, serve as sensors for reactive oxygen species (ROS) (Wrzaczek *et al.*, 2010). Some members of the DUF26 family were observed to be transcriptionally induced by ozone and pathogen attack, suggesting their relationship with ROS homeostasis and impact on biotic stress responses (Chen *et al.*, 2004; Wrzaczek *et al.*, 2010).

In *Arabidopsis*, most of the *CRK*s are located in tandem arrays on chromosome 4 and show a high level of sequence similarity, suggesting that several members of this family may be characterized by genetic redundancy. However, data collected so far indicate that some *CRK*s significantly affect plant development and stress responses. Two *CRK* genes, *CRK36* and *CRK45*, play antagonistic roles in abscisic acid-mediated pathways (Tanaka *et al.*, 2012; Zhang *et al.*, 2013b). The other two members of the DUF26 family, *CRK6* and *CRK7*, play a protective role in apoplastic oxidative stress triggered by ozone (Idänheimo *et al.*, 2014). Several members of the *CRK* family *(CRK4*, *CRK5*, *CRK6*, *CRK10*, *CRK11*, *CRK19*, *CRK20*, *CRK45*) are induced by salicylic acid (SA), which is a key molecule in the development of systemic acquired resistance (Czernic *et al.*, 1999; Chen *et al.*, 2003; Conrath, 2006; Zhang *et al.*, 2013a). Most results come from studies based on lines over-expressing *CRK*. Constitutive expression of *CRK5*, *CRK13*, and *CRK20* leads to increased tolerance to the virulent bacterial pathogen *Pseudomonas syringae* pv. *tomato* DC3000, which correlates with accumulation of SA and the activation of defence marker genes, *PR1*, *PR5*, and *ICS1* (Chen *et al.*, 2003; Acharya *et al.*, 2007; Ederli *et al.*, 2011). This suggests a role for *CRK*s in SA-mediated transduction pathways. Indeed, in the transcriptomic screen of the whole *CRK* family the expression pattern of many genes was shown to be regulated by SA. The *sid2* mutant (deficient in SA biosynthesis) and *npr1* mutant (impaired in signalling in response to SA) show downregulation of many *CRK*s, while in the *dnd1* mutant (producing elevated SA levels), increased expression of many DUF26 kinases was observed (Wrzaczek *et al.*, 2010). Previous reports show that dexamethasone-inducible over-expression of *CRK5* triggers the hypersensitive response-like cell death phenotype. By contrast, the same study (Chen *et al.*, 2003) did not find any considerably changed phenotypes in plants with constitutive expression of three other *CRK* genes (*CRK6*, *CRK10*, and *CRK11*). This suggests that *CRK5* in particular plays an essential role in response to pathogens and, in spite of its structural similarity to the other DUF26 members, does not possess any symptoms of genetic redundancy. However, the putative role of *CRK5* in plant development and abiotic stress acclimation has not been investigated before, and this issue is addressed in our study.

Our analysis revealed a strict correlation between *CRK5* knock-out and lower biomass production. It was accompanied by impaired stomatal conductance and elevated ROS level, which are known to interfere with photosynthetic efficiency (Farquhar and Sharkey, 1982; Foyer and Shigeoka, 2011). Moreover, the *crk5* mutant showed accelerated senescence, which was even more apparent under continuous darkness and oxidative stress. Quantitative PCR analysis showed a significantly increased expression level of several genes involved in ethylene and SA signalling in the mutant plants, suggesting the regulatory function of *CRK5* in maintaining hormonal balance in plant cells. The *crk5* plants also exhibited impaired acclimation to UV radiation, indicated by disrupted activity of ROS-scavenging enzymes and enhanced cell death. Transgenic lines with constitutive *CRK5* expression in the mutant background in most studies were able to revert the *crk5* phenotype, indicating an essential role of this gene in the regulation of growth, development, and abiotic stress acclimation.

## Materials and methods

### Plant material

**Fig. 1.**
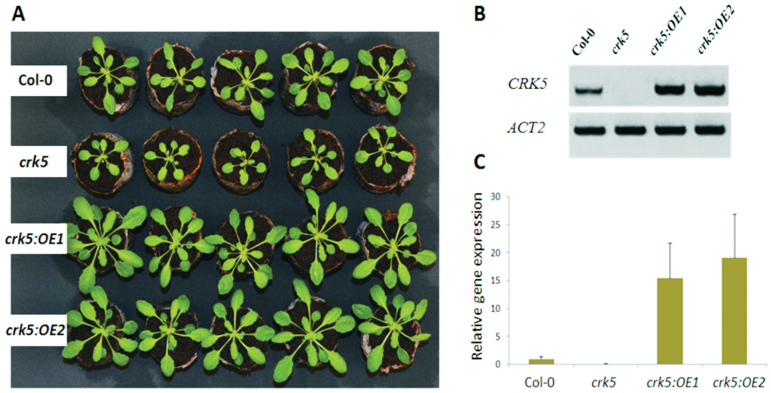
Phenotypic and genetic characterization of *crk5* recessive mutant and complementation lines. (A) Morphological phenotype of 5-week-old wild type, *crk5*, and two transgenic lines with constitutive expression of *CRK5* in the mutant background *crk5:OE1* and *crk5:OE2*. The *CRK5* transcript level was quantified in analysed genotypes using (B) reverse transcriptase PCR and (C) quantitative PCR.

All *Arabidopsis thaliana* plants used in this study were in the Columbia (Col-0) background. The T-DNA insertional mutant seeds of *crk5* (SALK_063519C) were obtained from the Nottingham Arabidopsis Stock Centre (Loughborough, UK) and confirmed by PCR (all primers are listed in Supplementary Table S1). They were backcrossed twice to the wild-type plants; homozygous *Arabidopsis* mutant seeds from the F5 generation were used for further studies. Quantitative PCR and reverse transcriptase PCR were performed to show the *CRK5* expression level (Fig. 1B,C).

For the generation of complementation lines, the open reading frame of *CRK5* was amplified using cDNA prepared from *Arabidopsis* (Col-0) as template. PCR products were purified with a gel extraction kit (Qiagen, Hilden, Germany), inserted into the entry vector using the pENTR/D-TOPO Cloning Kit (Invitrogen, Carlsbad, CA, USA), and verified by sequencing using M13 primers. The products were then cloned into the pGWB41 binary destination vector to allow expression from the cauliflower mosaic virus 35S promoter. The construct was introduced into *Agrobacterium tumefaciens* strain GV3101 and transformed into the *crk5* mutant line by the floral dip method, according to Bechtold *et al.* (2003). The transgenic lines were identified based on hygromycin selection. Quantitative PCR was performed to show the *CRK5* expression level. For the generation of lines for spatial localization of CRK5, the 1248bp promoter region of *CRK5* was used for PCR amplification and the pHGWFS7 vector was used as a destination vector.

### Growing conditions

The seeds underwent cold stratification for 3 days at 4°C and were then grown on peat with perlite (mixed in the ratio 3:1) or in Jiffy Pots (Jiffy Products, Oshkosh, WI, USA) in the growing room under the following conditions: short-day photoperiod (8h light/16h dark), temperature 22/18°C (day/night, respectively), relative humidity of 70±5%. Plants were grown under white fluorescent lamps at a light intensity of 100 μmol m^−2^ s^-1^.

### Stress treatment

For UV experiments, the UVC 500 Crosslinker (Hoefer Pharmacia Biotech, San Francisco, CA, USA) was used as a light source. It was equipped with three UV-B lamps (type G8T5E, Sankyo Denki, peak wavelength 306nm) and two UV-A lamps (type TL8WBLB, Philips, peak wavelength 365nm) and contained no UV-C to avoid necrotic responses of the plants. Plants were exposed to single radiation episode at the dose 1500 mJ·cm^−2^ and analysed up to 48h after stress treatment.

### Relative electrolyte leakage

The leaves were excised and transferred into 50mL Falcon tubes containing 35mL Milli-Q (Merc Millipore, Darmstadt, Germany) water. The relative electrolyte leakage was measured with a conductance meter (WTW, INOLAB Cond Level 1, Weilheim, Germany) and calculated as a ratio between the value obtained after 1h incubation and the total leakage evaluated after autoclaving the samples.

### Trypan blue staining of programmed cell death

Staining was performed as previously described (Koch and Slusarenko, 1990) with minor modifications. Leaves were submerged in stain solution (0.016% trypan blue, 8% phenol, 8% glycerol, 8% lactic acid, 65% ethanol), boiled for 3min, and incubated overnight. Then the samples were decolourized in bleaching solution (6M chloral hydrate). The stained tissues were observed under a light microscope and digital images of the leaves were captured using a fluorescent stereo microscope (Leica M165-FC; Leica Microsystems, Wetzlar, Germany). For measurements, one leaf was sampled (the seventh) from at least six different plants per genotype.

### Chlorophyll *a* fluorescence

Chlorophyll *a* fluorescence parameters were determined on whole rosettes using a pulse amplitude-modulated FluorCam 800 MF and the associated software (Photon Systems Instruments, Drasov, Czech Republic). Prior to measurements, the plants were kept in darkness for 30min to determine *F*_0_ and *F*_m_, and then exposed to 5min of actinic red light (90 μmol m^−2^ s^−1^) to determine *F*_t_ and *F*_m_ʹ. The actinic light was then switched off and after incubation with far-red light *F*_0_′ was determined. The effective photosystem II (PSII) quantum yield Y(II) = (*F*_m_′ − *F*_s_)/*F*_m_′, photochemical quenching *qP* = (*F*_m_ʹ− *F*_t_)/(*F*_m_ʹ − *F*_o_ʹ), and non-photochemical quenching NPQ = [(*F*_m_/*F*_m_ʹ) − 1] were determined as described earlier (Kramer *et al.*, 2004; Baker, 2008).

### Analysis of stomatal closure and density

To estimate the stomatal closure (µm) and density (number of stomata per mm^2^) abaxial epidermal strips from similarly developed leaves were analysed under a laser scanning confocal microscope. Stomata were visualized by chlorophyll autofluorescence in guard cells at an excitation wavelength of 488nm. Stomatal apertures were determined as the ratio of width to length using Image J analysis computer software (http://rsb.info.nih.gov/ij/). Mean values [± standard deviation (SD)] were derived from three individual leaves of three different plants. For each leaf, stomata were counted from six randomly chosen 0.312mm^2^ picture areas.

### Determination of H_2_O_2_ content

Plant tissue was homogenized in ExB buffer (containing 50mM HEPES pH 7.5 and 1mM EDTA) and centrifuged (14 000rpm, 10min, 4^o^C). Subsequently, 400 µL of supernatant was mixed with 400 µL of chloroform and methanol (2:1) solution and centrifuged (10 000rpm, 3min, 4^o^C). Next, 400 µL of supernatant was added to a solution containing 2.54mL HEPES buffer, 30 µL homovanillic acid (50mM) and 30 µL horseradish peroxidase (4 µM). After 10min of incubation, experimental data were acquired by the use of a Hitachi F-2500 spectrofluorometer (Schaumburg, IL, USA) at the excitation wavelength of 315nm. The resultswere calculated in nmol H_2_O_2_ per gram of dry weight, on the basis of the standard curve used for calibration.

### Restricted gas exchange assay

Plants were grown on one-half-strength Murashige and Skoog agar medium for 2.5 weeks. Then, the plates were transferred to continuous light exposure (180 µM m^−2^ s^−1^) and sealed with two layers of Parafilm M (Bemis, Neenah, WI, USA) to restrict gas exchange within Petri dishes. Photographs were taken before and 10 days after Parafilm application.

### Determination of water-use efficiency

Water-use efficiency (WUE) was determined as dry weight per unit of water used (mg dry weight·mL^–1^ water) by 4-week-old plants grown in the short-day photoperiod. Plants were grown in 50-mL tubes filled with perlite and soil (mixed in a ratio 2:1) and 35mL of water. Seeds were placed in a hole (approximately 1.5mm wide) made in the cap. After germination, the system was weighed. Plants were decapitated after 4 weeks’ growth and dried for 3h at 105°C to determine their dry weight. Then, each tube was weighed to estimate the water loss.

### Gas exchange analysis

Gas exchange parameters were measured using Portable Gas Exchange Fluorescence Systems GFS-3000 (Walz, Effeltrich, Germany) as described before (Wituszynska *et al.*, 2013). The regression line slopes were compared using the procedure from Wonnacott and Wonnacott (1990) based on the use of a mute variable D.

### Pigment content analysis

Pigment content analysis was performed as previously described (Wituszynska *et al.*, 2013). Results are given as the peak area per microgram of dry weight.

### Diaminobenzidine staining

*In situ* detection of H_2_O_2_ in mature *Arabidopsis* rosette leaves was made by staining with 3,3ʹ-diaminobenzidine (DAB). After decapitation leaves were submerged in DAB solution (1mg DAB/mL solution, 0.05% v/v Tween 20, 10mM Na_2_HPO_4_, pH 3), vacuum infiltrated for 5min, and incubated at room temperature with gentle shaking for 4h. Then, the staining solution was replaced with bleaching solution (ethanol, acetic acid, and glycerol in a ratio of 3:1:1), samples were put in boiling water (~95°C) for 15min, and visualized under the microscope.

### Determination of antioxidant enzymes activities

The activities of catalase (CAT), superoxide dismutase (SOD) and ascorbate peroxidase were measured as described previously (Gawronski *et al.*, 2014). All measurements were performed using a UV-Vis Multiskan GO Microplate Spectrophotometer (Thermo Scientific, Waltham, MA, USA).

### Determination of ethylene content

The analysis of ethylene emission was made on 5-week-old plants using a gas chromatograph (GC 6890+ with FID detector, Agilent, Santa Clara, CA, USA). Prior to the measurement, plants were transferred to 100mL tubes, closed to prevent air exchange with the environment, and incubated for 72h. This was a compulsory step to generate a detectable gas chromatography signal. Measurements were made in at least four biological and three technical replicates. The results are expressed as microlitres of ethylene per litre of air (ppm) per dry weight. The determination of ethylene content was calculated from the standard curve created on the basis of two standards with known ethylene concentration (1 ppm and 3 ppm).

### Real-Time PCR

RNA isolation, and preparation of cDNA and PCR amplifications were performed as described previously (Gawronski *et al.*, 2014). RNA purity was checked with PCR with *ACT2* specific primers (Supplementary Table S1). For each experiment, the best reference gene was selected from the list of two genes: *UPL7* and *YLS8* (Czechowski *et al.*, 2005) using the geNorm algorithm (Vandesompele *et al.*, 2002).

### β-glucuronidase histochemical staining

Two-week-old seedlings (carrying the construct *CRK5 promoter::GUS*) were immersed in staining solution made fresh immediately prior to use (80mM phosphate buffer pH 7, 20% methanol, 0.04% Triton X-100, 1mM X-Gluc). This was followed by 1h vacuum infiltration and incubation overnight at 37°C. After removal of staining solution, seedlings were washed with several changes of 70% ethanol until the tissue became clear of chlorophyll and the blue colour resulting from the conversion of X-Gluc became visible.

### Determination of SA content

Determination of SA was performed as described by Meuwly and Métraux (1993). 2-methoxybenzoic acid and 3-hydroxybenzoic acid were used as the internal standards. SA was eluted on a Luna 5uC18(2)100A 150×4.6mm column (Phenomenex, Torrance, CA, USA) at 30°C for 15min using a Shimadzu HPLC System (Columbia, MD, USA). A low pressure gradient system was used with 20 mmol phosphate buffer (pH 2.5; adjusted with 8M HCl) and acetonitrile (75:25; v/v) at the flow rate of 1mL min^-1^. The results are expressed as micrograms of SA per gram of dry weight.

### Statistical analysis

The statistical analyses of chlorophyll *a* fluorescence and gas exchange were performed in the “R” programming environment version 2.12.1 using stats packages. Statistical analyses of relative ion leakage; stomatal aperture; WUE and the content of chlorophyll, SA, ethylene, H_2_O_2-_, and antioxidant enzymes were performed using GraphPad Prism 5.0 (GraphPad Software, La Jolla, CA, USA).

## Results

### Regulation of WUE

**Fig. 2.**
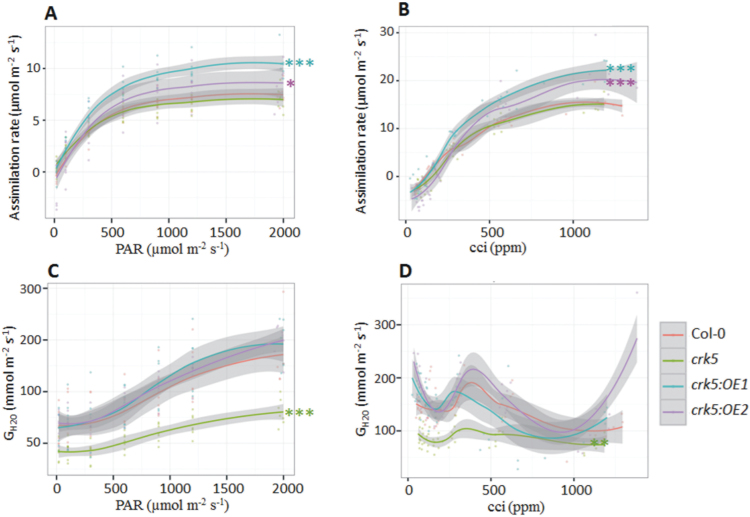
Gas exchange analysis. The curves describe the relationship between foliar CO_2_ assimilation and (A) increasing values of photosynthetically active radiation (PAR) and (B) growing CO_2_ concentration. The analysis also involved a calculation of (C) water vapour conductance (G_H2O_) under increasing values of PAR and (D) growing CO_2_ concentration. The analysis was performed on 3.5-week-old plants, with nine replicates (n = 9). Grey shades indicate 95% confidence intervals. Asterisks indicate a significant difference of the regression line slopes at level *P < 0.05, **P < 0.01, *** *P <* 0.001.

The rate of assimilation in the analysed plants was estimated depending on variable light intensity (10–2000 µmol m^-2^ s^-1^) and CO_2_ concentration (20–1400 ppm). During the modulated light experiment, all the lines displayed a maximum CO_2_ uptake rate at light intensity ranging from 1700 to 1800 μmol m^−2^ s^−1^. The *crk5* mutant plants did not show significant differences in CO_2_ uptake compared to wild type; however, both transgenic lines with constitutive *CRK5* expression in the mutant background displayed markedly elevated assimilation capacity, especially under high and saturating light (particularly the *crk5*:OE1 plants, reaching values >10 μmol CO_2_ m^−2^ s^−1^) (Fig. 2A). This characteristic pattern was also observed when analysing the assimilation rate with growing intercellular CO_2_ concentration. Although the mutant plants showed no difference compared to wild type, both transgenic lines exhibited substantially enhanced CO_2_ uptake exceeding 20 μmol m^−2^ s^−1^ (Fig. 2B), suggesting positive correlation between increased *CRK5* expression and higher capacity of CO_2_ assimilation. This was reflected in the morphological phenotype of the analysed lines. The 5-week-old transgenic plants were easily distinguished by their enhanced biomass production (Fig. 1A), while the mutant plants, although not impaired in CO_2_ uptake capacity (Fig. 2A,B), showed a decreased growth rate and lower biomass production (Fig. 1A). This phenotype may result from lower WUE. The *crk5* plants produced approximately 20% less dry mass per 1mL of water utilized compared with the wild type, while complementation lines managed to reverse this phenotype (Supplementary Fig. S1).

Reduced WUE in the *crk5* plants correlated with significantly impaired water vapour conductance (G_H2O_), calculated from the ratio of transpiration to water saturation inside the leaf. A substantial decline was observed already under low light conditions, and with growing light exposure the G_H2O_ value in the wild type rose more quickly compared with the mutant plants, making the observed differences even more striking (1.7-fold higher G_H2O_ in the wild type compared with *crk5* at saturating light) (Fig. 2C). Interestingly, the transgenic lines used in the study did not show enhanced G_H2O_ compared to wild type, suggesting that, although the lack of *CRK5* expression significantly impaired stomatal conductance, constitutive expression of this gene did not improve the capacity of plants to regulate this process. The shape of the G_H2O_ curve in the mutant plants also showed a significant difference with the growth of intercellular CO_2_ concentration. In the wild type and complementation lines the curve showed initially decreasing G_H2O_ values, followed by their rapid growth up to the plateau phase at intercellular CO_2_ concentration (Ci) of 350–400 ppm. Opposite to this, the *crk5* line hardly showed any oscillations during the modulated CO_2_ and was not able to accelerate stomatal conductance under growing CO_2_ at any measuring point (Fig. 2D), suggesting that stomatal conductance in the mutant plants is not susceptible to variable CO_2_ concentration.

**Fig. 3.**
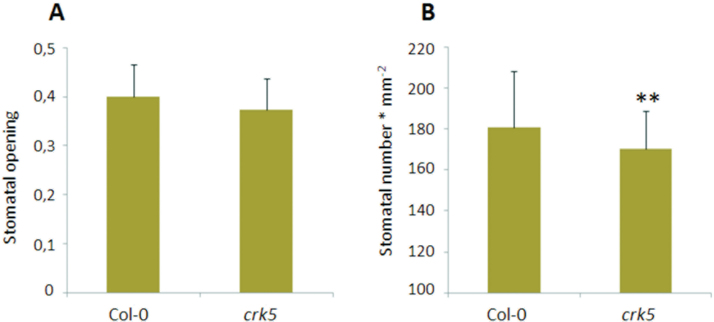
Stomatal aperture in 4-week-old plants. (A) Stomatal number calculated per 1mm^2^. (B) The level of stomatal opening measured as a ratio of width to length of stomata under growing conditions. Mean values (±SD) were derived from three individual leaves of five different plants. The number of stomata was counted from six randomly chosen 0.312mm^2^ picture areas (n = 90), while stomatal opening was measured from four randomly chosen 0.312mm^2^ picture areas (n = 60). Asterisks indicate a significant difference according to the *t*-test at level *P* < 0.001 (**)

Based on the results from gas exchange analysis showing impaired water vapour conductance in *crk5* not only under high light exposure but also in growing conditions, the stomatal aperture and density in the mutant plants were investigated. No significant difference in stomatal opening was found (Fig. 3A); however, microscopic analysis revealed a reduced number of stomata in *crk5* leaves (170±18.7mm^−2^) in comparison to wild type (181±27.3mm^−2^) (Fig. 3B). Nevertheless, the observed difference in stomatal aperture was not so striking or relevant to fully explain the disrupted water vapour conductance in mutant plants. Because a decreased biomass production in *crk5* did not result from changes in CO_2_ assimilation capacity but was accompanied by lower WUE, the next step was to check if *crk5* is affected in the photochemical processes during the light phase of photosynthesis, which are dependent on light-induced catalytic water splitting to produce ATP and NADPH.

### Chlorophyll *a* fluorescence

**Fig. 4.**
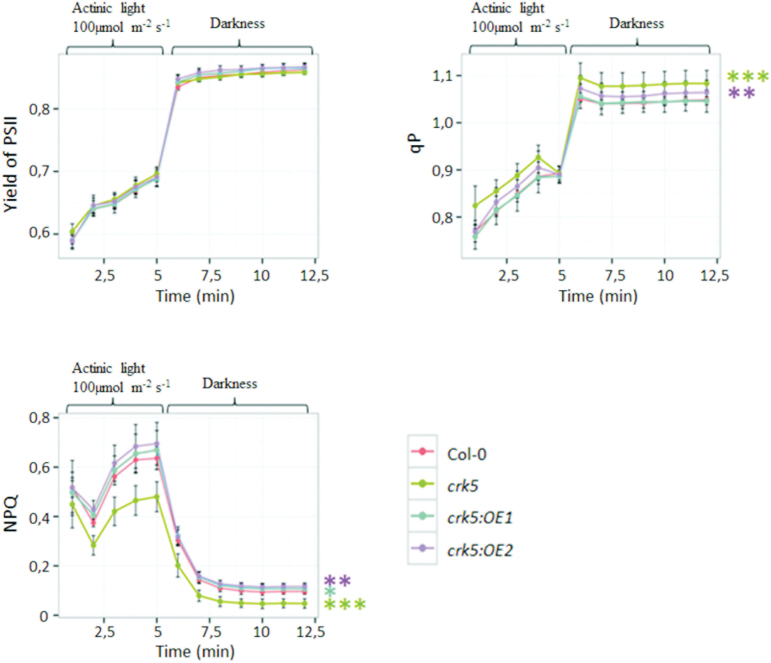
Photosynthetic parameters determining the activity of PSII calculated from chlorophyll *a* fluorescence. (A) Effective quantum yield of PSII (Y_PSII_). (B) photochemical quenching (*qP*). (C) Non-photochemical quenching (NPQ). Data represent mean values of 12 plants ± SD (n = 12). Asterisks indicate a significant difference of the regression line slopes at level *P < 0.05, **P < 0.01, ***P < 0.001.

To investigate the role of *CRK5* in the regulation of photosynthesis in *Arabidopsis*, chlorophyll *a* fluorescence in the *crk5* mutant, the wild type, and the complementation lines was tested. Measurements of different fluorescence parameters in analysed plants did not reveal changes in the effective quantum efficiency of PSII (yield of PSII), which reflects the proportion of light absorbed by chlorophyll associated with PSII that is used in photochemistry and, thus, is often used to calculate linear electron transport rate (Fig. 4A). Nevertheless, the *crk5* showed markedly enhanced photochemical quenching (*qP*) (Fig. 4B), suggesting that the plastoquinone was more oxidized in plants lacking *CRK5* (as indicated by lower 1 – *qP*). The redox state of the plastoquinone pool triggers many light-stimulated physiological responses of plants. Oxidation of the plastoquinone pool is often caused by singlet oxygen generated in PSII or results from smaller photosynthetic antenna size, suggesting limited light-harvesting capacity of *crk5*. However, the mutant plants exhibited a significant reduction in the efficiency of non-photochemical quenching (NPQ), indicating that they were impaired in thermal dissipation of excess excitation energy (Fig. 4C and Supplementary Fig. S2). This process prevents photo-oxidative damage of PSII, thus avoiding photoinhibition, which can decrease plant fitness and productivity. The *crk5* mutant showed the most apparent NPQ decrease during the first 5min of chlorophyll *a* fluorescence measurement, which corresponds to the time when the plants were exposed to actinic red light. At the end of the measuring programme, when the light was switched off and the plants were incubated in the darkness, the observed differences in NPQ between mutant plants and wild type were not as striking (Fig. 4C). Complementation lines reversed this phenotype and even showed slightly enhanced capacity for thermal dissipation of light energy.

### Regulation of leaf senescence

**Fig. 5.**
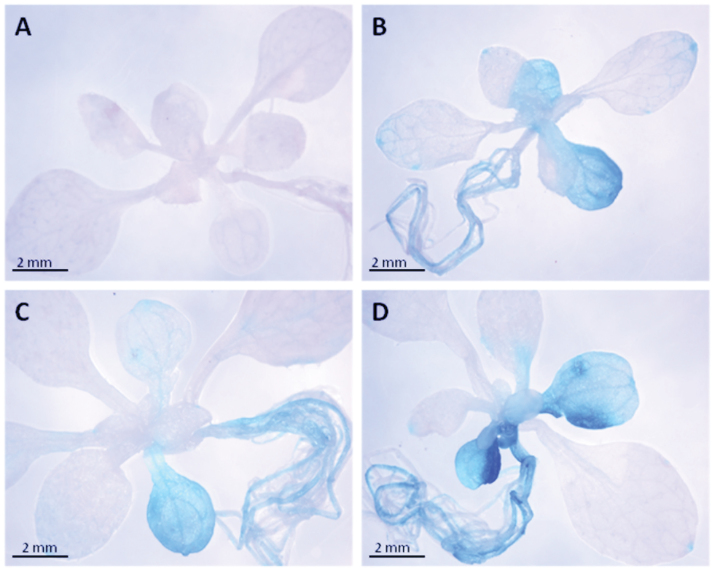
Histochemical GUS staining for spatial localization of *CRK5* in 2.5-week-old seedlings. (A) Col-0 control plants. (B, C, D) *CRK5 promoter::GUS* transgenic plants.

**Fig. 6.**
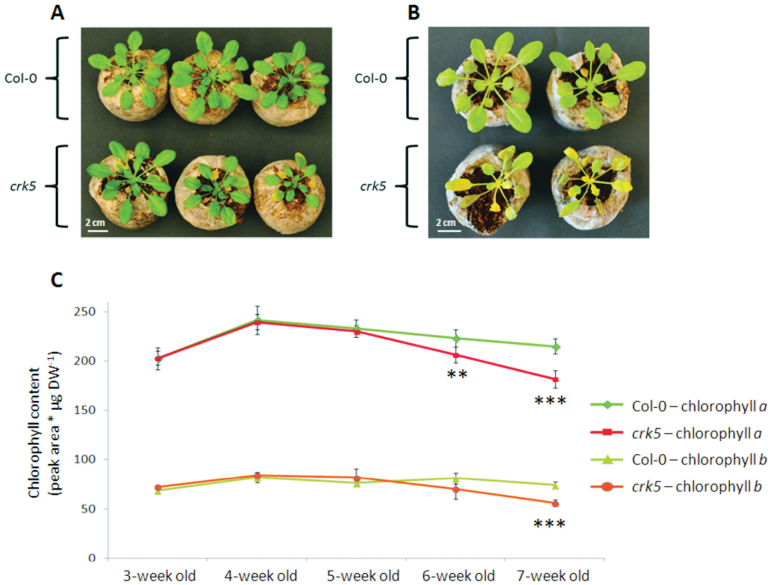
Accelerated senescence phenotype in *crk5* plants. (A) Morphological phenotype of accelerated senescence in *crk5.* Pictures of 5.5-week-old plants grown in a short day photoperiod. Scale bar = 2cm. (B) Morphological phenotype of accelerated senescence in *crk5* under continuous darkness. Pictures of 4-week-old plants grown in a short day photoperiod and kept in darkness for 6 days. Scale bar = 2cm. (C) Age-dependent chlorophyll *a* and *b* content in plants grown in a short day photoperiod. Mean values (±SD) are derived from six plants (n = 6). Asterisks indicate a significant difference according to the *t*-test at level *P < 0.05, **P < 0.01, ***P < 0.001.

Spatial localization studies made on 2-week -old seedlings showed the most visible *CRK5* expression, especially in the cotyledons, hypocotyls, and roots (Fig. 5 and Supplementary Fig. S3). It corresponded to the phenotype of *crk5* mutant, which showed rapid cell death in cotyledons shortly after the development of true leaves during the growth of young seedlings. The early decomposition of cotyledons correlated with visible symptoms of accelerated senescence under growing conditions, manifested by premature leaf yellowing (Fig. 6A), and substantially slowed down biomass production (Fig. 1A). Physiological changes leading to enhanced *crk5* aging were more induced in plants kept under continuous darkness conditions (Fig. 6B) or in a low CO_2_ concentration (Supplementary Fig. S4). To study the age-dependent profile of the life cycle, chlorophyll *a* and *b* contents were measured at different stages of plant growth. No difference was observed in younger plants up to 5 weeks’ age. However, a significant chlorophyll decline started in 6-week-old *crk5* compared to wild type (Fig. 6C). In 7-week-old mutant plants, the observed differences were even more apparent.

**Fig. 7.**
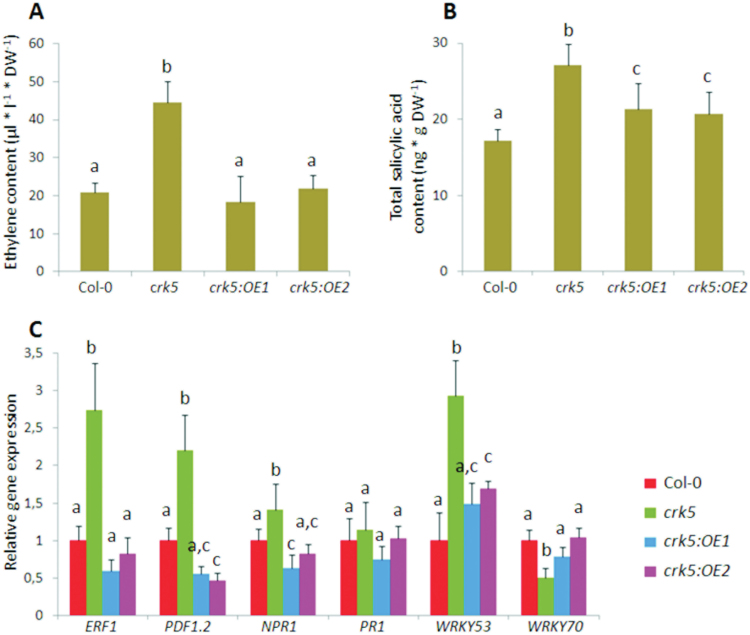
Alterations in salicylic acid and ethylene signalling in 5-week-old plants. Foliar accumulation of (A) salicylic acid and (B) ethylene under growing conditions. Mean values (±SD) are derived from nine plants (n = 9). (C) Transcript profiling of salicylic acid-, ethylene- and senescence-responsive genes in analysed genotypes. Data show relative expression normalized to the wild type and represent average values ±SD. Different letters above the bars indicate a significant difference at *P* < 0.005 (Tukey’s test).

To search for mechanisms responsible for enhanced leaf aging in *crk5*, the foliar level of ethylene was measured. In this study, the accumulation of ethylene was significantly elevated in 5-week-old *crk5* plants (Fig. 7A) and correlated with higher transcript abundance of two genes involved in ethylene signalling, ethylene response factor 1 (*ERF1*) and plant defensin 1.2 (*PDF1.2*) (Fig. 7C). Complementation lines managed to revert the mutant phenotype, suggesting that *CRK5* might negatively regulate ethylene signalling pathways during senescence processes.

The analysis of the *CRK5* promoter showed a significant enrichment in W-Box *cis*-regulatory elements (Supplementary Fig. S5), which play a role in the SA-responsive expression pattern. Thus, this study also involved the measurements of SA level in plant leaves and revealed its increased accumulation in 5-week-old mutant plants. However, this observation was not fully specific, because an elevated SA content was also found in complementation lines with constitutive *CRK5* expression (Fig. 7B). Increased SA accumulation in the mutant plants correlated with higher transcript abundance for nonexpressor of pathogenesis-related proteins 1 (*NPR1*), which acts as an SA receptor and is essential for the activation of SA-dependent defence genes. However, a well-known marker gene involved in SA signalling, pathogenesis-related gene 1 (*PR1*), showed no statistically significant differences in any of the analysed plant lines (Fig. 7C). Interestingly, the expression of two SA-responsive transcription factors, *WRKY53* and *WRKY70*, was found to be substantially altered in the analysed plants. These two genes were previously widely described for their positive and negative regulatory role in senescence, respectively. This study showed a nearly 3-fold *WRKY53* increase in 5-week-old *crk5* plants, while the expression of *WRKY70* was decreased (Fig. 7C).

### Regulation of acclimatory responses to UV radiation

**Fig. 8.**
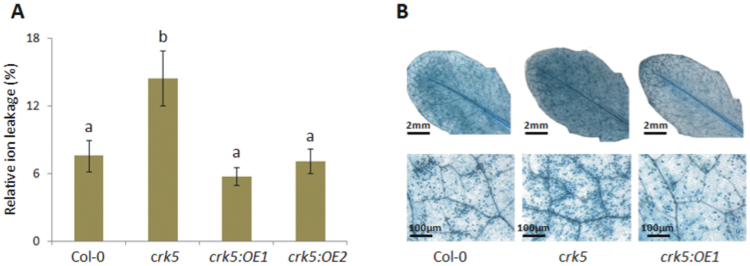
The rate of membrane damage and cell death in analysed genotypes and after 48h from UV episode at a dose of 1500 mJ cm^-2^. (A) Relative cellular electrolyte leakage. Mean values (±SD) are derived from 12 plants (n = 12). Different letters above the bars indicate a significant difference at *P* < 0.005 (Tukey’s test). (B) Trypan blue staining for the detection of cell death.

**Fig. 9.**
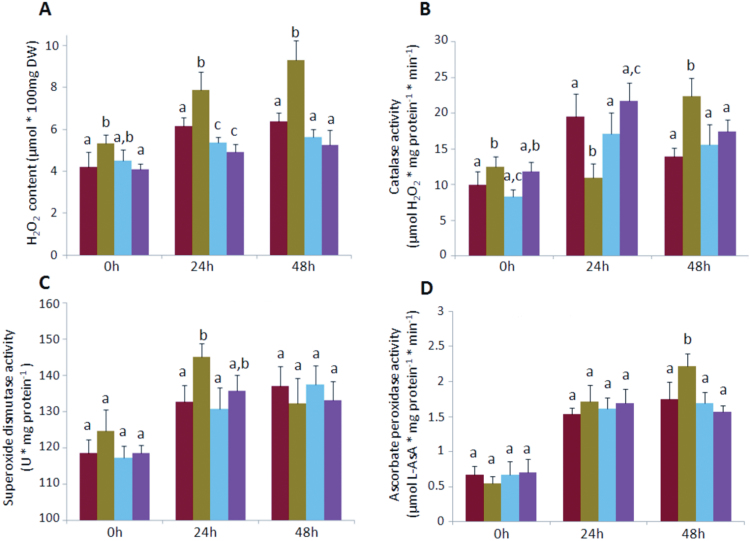
Foliar content of H_2_O_2_ and activity of ROS-scavenging enzymes under growing conditions and 24 and 48h after UV radiation at a dose of 1500 mJ cm^-2^. (A) H_2_O_2_ content assessed by histochemical activity of horseradish peroxidase. (B, C, D) The activity of oxidoreductive enzymes: (B) catalase, (C) superoxide dismutase, and (D) ascorbate peroxidase. Mean values (±SD) are derived from nine plants (n = 9). Different letters above the bars indicate a significant difference at *P* < 0.005 (Tukey’s test).

The analysis of microarray data collected from *Arabidopsis* eFP browser (http://bar.utoronto.ca/efp/cgi-bin/efpWeb.cgi) showed that, among different stress treatments, *CRK5* exhibited the most significant upregulation in response to UV radiation (Supplementary Fig. S6). Because of this, the potential role of this gene in the acclimation to UV was explored. Here, plants were treated with a combination of UV-A and UV-B spectra but not UV-C to avoid necrotic responses. The results from ion leakage demonstrated a considerably disrupted membrane stability in *crk5* plants, as indicated by their 2-fold increase in electrolyte outflow compared to wild type (Fig. 8A). Enhanced cell death in the leaves of mutant plants was also confirmed by trypan blue staining (Fig. 8B). Complementation lines reverted this phenotype, suggesting a role for *CRK5* in the regulation of cell death. The protective activity of this gene in stress acclimation may be related to the regulation of ROS homeostasis. As shown above (Fig. 4C), thermal dissipation of excess excitation energy was affected in plants lacking *CRK5* expression, which makes them more susceptible to photo-oxidative damage owing to the production of excessive singlet and triplet states of chlorophyll inside the chloroplasts. Indeed, the results showed that *crk5* accumulated more ROS. A significantly increased level of H_2_O_2_ was found in the mutant plants under growing conditions and, after a UV-driven oxidative burst, they displayed an even more striking differences with more than 40% higher accumulation of H_2_O_2_ (9.28±0.95) compared to wild type (6.38±0.39) (Fig. 9A and Supplementary Fig. S7). Complementation lines reverted the mutant phenotype and even showed decreased H_2_O_2_ production, compared to wild type, 24h after UV exposure (Fig. 9A). Disrupted ROS balance in *crk5* correlated with alterations in the activities of ROS-scavenging enzymes, CAT, and SOD. A sharp increase in SOD level in the mutant plants was observed 24h after UV exposure (Fig. 9B), whereas CAT activity was significantly lower (Fig. 9C), which together contributed to a rapid increase of H_2_O_2_ concentration shortly after stress treatment. The mutant plants showed a substantial delay in the activation of H_2_O_2_ scavenging enzymes. Although a significant upregulation of CAT and ascorbate peroxidase were found in *crk5* 48h after UV exposure (Fig. 9B,D), higher oxidative damage inside the cells of the mutant plants up to this time resulted in visible symptoms of enhanced cell death. Collectively, these data suggest that CRK5 plays an essential role in ROS signalling pathways, WUE, senescence, and cell death.

## Discussion

Previous reports showed that constitutive expression of *CRK5* led to increased resistance to *P.syringae* and enhanced leaf growth in transgenic plants (Chen *et al.*, 2003; Chen *et al.*, 2004), while steroid-induced over-expression of this gene triggered hypersensitive response-like cell death (Chen *et al.*, 2003). By contrast, in the same study, no altered phenotype was found in plants with constitutive expression of three other *CRK* genes analysed (*CRK6*, *CRK10*, and *CRK11*), suggesting that *CRK5* does not show genetic redundancy with other *CRK* members and appears to be an interesting candidate for the regulation of important developmental and acclimatory processes in plants. Here, a regulatory function of this gene was investigated by functional characterization of *crk5* mutant plants, supported by the generation of transgenic lines with constitutive *CRK5* expression in the mutant background. The *crk5* plants showed a clear phenotype during growth, development, and stress response, while complementation lines, in most studies, were able to recover the wild-type phenotype.

This study indicates that *CRK5* is a negative regulator of cell death, which affects both senescence and acclimatory processes. The most visible expression of this gene in young plantlets, monitored by assaying for GUS product (β-glucuronidase), was found in the cotyledons (Fig. 5 and Supplementary Fig. S3). Interestingly, this expression corresponded with the phenotype of *crk5* plants, showing a rapid cell death in cotyledons shortly after the development of true leaves during the growth of young seedlings, and subsequent accelerated senescence (Fig. 6A,C). This phenotype appeared even more striking under continuous darkness and after deprivation of foliar gas exchange leading to rapid CO_2_ consumption (Supplementary Fig. S4). In these conditions, *crk5* mutant plants displayed enhanced cell death similar to catalase-deficient plants (Hackenberg *et al*., 2013; Kerchev *et al.*, 2014). A similar effect was also previously reported in *lsd1* mutant plants, which displayed runaway cell death after artificial blocking of stomatal pores and gas exchange by smearing lanolin on the lower surface of the leaves (Mateo *et al.*, 2004; Li *et al.*, 2013). It was suggested that lesion formation in *lsd1* under these conditions resulted from the impairment of stomatal conductance and lower activity of antioxidant enzymes unable to scavenge excessive ROS production (Mateo *et al.*, 2004; Muhlenbock *et al.* 2008).

Similar to *lsd1*, the *crk5* plants showed altered stomatal conductance and impaired H_2_O_2_ decomposition, leading to oxidative damage especially when challenged with ROS-triggering stress stimuli (Fig. 2C, Fig. 9A, and Supplementary Fig. S7). Impaired ROS homeostasis in *crk5* most probably contributed to its accelerated senescence phenotype, leading to an accumulation of senescence-related hormone, ethylene, and subsequent upregulation of *ERF1* and *PDF1.2* (Fig. 7A,C), which participate in ethylene signalling pathways (Overmyer *et al.* 2003; Leon-Reyes *et al.*, 2010; Zarei *et al.*, 2011; Cheng *et al.*, 2013). Moreover, the *crk5* plants showed a significant accumulation of SA (Fig. 7B), a stress-related hormone that has been previously linked to hypersensitive response and systemic acquired resistance (Alvarez, 2000; Lu, 2009), as well as to responses to high light, drought, salt, UV-B, and many others (Yuan and Lin, 2008; Miura and Tada, 2014). Bioinformatic studies show that a characteristic feature of the *CRK5* gene is an unusually large number of W-Box elements (TTGAC) in the promoter region (Supplementary Fig. S5). W-boxes are well known *cis*-regulatory elements recognized by WRKY transcription factors (Eulgem *et al.*, 2000), which are involved in the transcriptional regulation of genes associated with SA transduction pathways (Ulker and Somssich, 2004; Yu *et al.*, 2001). In this study, increased SA accumulation in the *crk5* plants correlated with upregulation of *NPR1* (Fig. 7C), which acts as an SA receptor and is essential for the activation of SA-dependent defence genes (Wu *et al.*, 2012). It suggests that *CRK5* is a component that functions downstream of SA and has a regulatory effect on ROS balance. An elevated concentration of SA in the mutant plants might contribute to accelerated senescence *via* a regulatory loop involving WRKY transcription factors.

In this study, two *WRKY* genes, *WRKY53* and *WRKY70*, were selected for transcriptomic analyses, as they were previously widely reported as positive and negative regulators of senescence, respectively (Ulker *et al.*, 2007; Miao *et al.*, 2008; Zentgraf *et al.*, 2010; Besseau *et al.*, 2012). Interestingly, transcript abundance of *WRKY70* was substantially lower in 5-week-old *crk5* (Fig. 7C), while the age-dependent *WRKY53* expression pattern showed a significant upregulation particularly in 6-week-old mutant plants. However, a higher expression level of this marker gene was also observed in *crk5* at earlier age stages analysed, compared to wild type and complementation lines (Supplementary Fig. S8), suggesting that processes responsible for the accelerated senescence phenotype in *crk5* begin relatively early in plant growth. Experiments performed by Miao *et al.* (2008) clearly demonstrate that *WRKY53* expression is induced by H_2_O_2_. Therefore, physiological inability of *crk5* plants to scavenge excessive H_2_O_2_, which leads to its accumulation even under growing conditions, may be one of the reasons for *WRKY53* upregulation and subsequent acceleration of senescence.

This study showed that *CRK5* had a positive impact on biomass production (Fig. 1), which is consistent with previously reported data showing enhanced leaf growth in plants with constitutive *CRK5* expression (Chen *et al.*, 2003). This phenotype might result from the regulatory effect of this gene on stomatal conductance (Fig. 2C), since limitations to photosynthesis and growth have already been linked with decreased transpiration (Katul *et al.*, 2010; Lawson and Blatt, 2014). The *crk5* plants exhibited completely deregulated water vapour conductance, while complementation lines managed to revert this phenotype and showed slightly higher capacity for stomatal conductance under high light, compared to wild type; however, these differences were not statistically important. Impaired stomatal conductance in the mutant line contributed to impaired WUE (Supplementary Fig. S1). However, this phenotype did not correlate with enhanced stomatal closure in *crk5* (Fig. 3A). Although the stomatal density in the mutant plants was decreased (Fig. 3B), the difference was not so apparent to explain this phenomenon. The transgenic *Arabidopsis* plants used for spatial localization studies also showed significant GUS activity in roots (Fig. 5 and Supplementary Fig. S3). Therefore, it cannot be excluded that *CRK5* is expressed in these tissues and might play a positive role in water and nutrient uptake from the soil, e.g. by regulation of root pressure. That could also partly explain accelerated senescence in *crk5* mutant plants, because it was previously shown that nutritional deficiency is related to enhanced leaf aging (Crafts-Brandner, 1992; Thomas and de Villiers, 1996; Kobayashi *et al.*, 2013). However, it needs detailed investigation to verify this hypothesis, with an analysis of root architecture and physiology.

Impaired stomatal conductance in *crk5* presumably entails a lower transpiration rate and subsequently limited cooling capacity. These factors could potentially lead to overheating of *crk5* plants, especially when grown under high temperatures or high light exposure. It cannot be excluded that a lower transpiration rate forced the mutant plants to reduce the conversion of light energy to heat during photosynthesis, as indicated by significantly lower NPQ values (Fig. 4C and Supplementary Fig. S2). NPQ is an essential mechanism required to protect all PSII reaction centres against photodamage by high light. The molecular mechanism of this process is based on quenching of singlet excited state chlorophylls via enhanced internal conversion to the ground state, thus harmlessly dissipating excess excitation energy as heat through molecular vibrations (Muller *et al.*, 2001). Thus, on the one hand, a decreased NPQ in *crk5* might be an essential factor protecting the plants from overheating due to impaired transpiration, but, on the other hand, a reduced capacity for thermal energy dissipation in *crk5* may lead to enhanced ROS production within the reaction centres in the chloroplast and subsequent photo-oxidative damage. This damage was clearly demonstrated by investigating pigment distribution in the chloroplasts of the analysed plants after UV episode (Fig. S9). These conditions triggered an enhanced oxidative burst in the mutant plants, indicated by an increased ratio of zeaxanthin to violaxanthin, compared to wild type, which is an indicative of an enhanced xanthophyll cycle. This mechanism plays an important role in the protection against oxidative stress by preventing the over-excitation of photosystems (Havaux *et al.*, 2000; Latowski *et al.*, 2011). An upregulated xanthophyll cycle in *crk5* plants might result from a reduced NPQ as an alternative way of protecting against photo-oxidative damage.

The study on plant acclimation to UV radiation further supported a role for *CRK5* as a regulator of ROS homeostasis. The *crk5* plants showed significantly greater susceptibility to UV, manifested by significant membrane damage and an increased level of cell death (Fig. 8A,B). The observed stress-induced phenotype was accompanied by markedly elevated H_2_O_2_ production (Fig. 9A). The significant upregulation of SOD 24h after UV radiation (Fig. 9B) suggests that a toxic superoxide radical might also have been overproduced in the mutant plants, further promoting cell damage. Significantly elevated zeaxanthin accumulation in the UV-treated *crk5* indicates that the signal transduction pathways leading to the observed changes also involve a chloroplast oxidative burst, presumably due to impaired NPQ and disrupted quenching of excited singlet and triplet states of chlorophyll (Young and Frank, 1996; Baroli and Niyogi, 2000). It suggests that the function of *CRK5* in the acclimation to UV may involve chloroplast retrograde signalling.

Taken together, *CRK5* appears to be a negative regulator of cell death and controls multiple processes, including not only the previously reported biotic stress response (Chen *et al.*, 2003), but also senescence, WUE, and acclimation to UV radiation. It presumably acts in a ROS-dependent manner and is probably involved in retrograde signalling from chloroplasts. A large number of W-Box elements in the promoter of this gene makes it a good candidate to participate in signalling pathways downstream of SA.

## Supplementary data

Fig. S1. Water-use efficiency (WUE) in analysed genotypes. WUE was calculated as mg of dry weight of 5-week-old plants per 1mL of water consumed by plants. Mean values (±SD) are derived from 12 plants (n = 12). Different letters above the bars indicate a significant difference at *P* < 0.005 (Tukey’s test).

Fig. S2. Representative pictures of chlorophyll *a* fluorescence parameters. The panels display false colour images of non-photochemical quenching (NPQ) of 4-week-old *Arabidopsis* rosettes. Scale bar = 2cm.

Fig. S3. Histochemical GUS staining in the tissues of *CRK5 promoter::GUS* transgenic plants. The highest GUS expression was found in cotyledons (A, B) and roots (C, D).

Fig. S4. Morphological phenotype of accelerated senescence in *crk5* after restriction of gas exchange. The plates with 2-week-old seedlings were transferred to a continuous light exposure (180 µM m^−2^ s^−1^) and taped with two layers of Parafilm to restrict gas exchange within Petri dishes. Photographs of four separate plates were taken before and 10 days after Parafilm application and combined as a single figure.

Fig. S5. Distribution of W-Box *cis*-regulatory elements in *CRK5* promoter region. These motifs are recognized and bound by WRKY transcription factors and activated via SA-mediated pathways.

Fig. S6. Analysis of *CRKs* expression in response to abiotic stress factors. The data were collected from eFP browser (http://bar.utoronto.ca/efp/cgi-bin/efpWeb.cgi) microarray results showing the level of CRKs transcription under different stress stimuli (cold, salt, drought, UV, wounding, heat, osmotic, and oxidative stress) All results of the phenotyping were normalized and integrated into the heat-map.

Fig. S7. Foliar content of H_2_O_2_ under growing conditions and after a UV radiation episode. H_2_O_2_ content assessed by DAB staining.

Fig. S8. Age-dependent transcript abundance for senescence marker gene *WRKY53* in analysed genotypes. Data show relative *WRKY53* expression normalized to 3-week-old wild type (Col-0) plants and represent average values ± SD. Different letters above the bars indicate a significant difference at *P* < 0.005 (Tukey’s test).

Fig. S9. Analysis of pigment content under growing conditions and 48h after a UV radiation episode. Data show relative pigment content normalized to untreated wild-type (Col-0) plants and represent average values ± SD. Different letters above the bars indicate a significant difference at *P* < 0.005 (Tukey’s test).

Table S1. Primers used in this study.

Supplementary Data

## Abbreviations:

CAT: catalase
CRK: cysteine-rich receptor-like kinase
DAB: 3,3ʹ-diaminobenzidine
G_H20_: water vapour conductance
NPQ: non-photochemical quenching
PSII: photosystem II
RLK: receptor-like protein kinases
ROS: reactive oxygen species
SA: salicylic acid
SD: standard deviation
SOD: superoxide dismutase
WUE: water-use efficiency.
