# Supplementary material for: Cysteine-rich receptor-like kinase CRK5 as a regulator of growth, development, and ultraviolet radiation responses in Arabidopsis thaliana
Source: J Exp Bot. 2015 May 12;66(11):3325–37. doi: 10.1093/jxb/erv143 (PMC4449547; doi:10.1093/jxb/erv143)
Supplement: Supplementary Data [file supp_66_11_3325__index.html]

Cysteine-rich receptor-like kinase CRK5 as a regulator of growth, development, and ultraviolet radiation responses in Arabidopsis thaliana — Cysteine-rich receptor-like kinase CRK5 as a regulator of growth, development, and ultraviolet radiation responses in Arabidopsis thaliana — Supplementary Data 

# Cysteine-rich receptor-like kinase CRK5 as a regulator of growth, development, and ultraviolet radiation responses in *Arabidopsis thaliana*

## Supplementary Data

Data files

**Files in this Data Supplement:**

- Supplementary Data - Supplementary Data
