# Supplementary material for: Cysteine-rich receptor-like kinase CRK5 as a regulator of growth, development, and ultraviolet radiation responses in Arabidopsis thaliana
Source: J Exp Bot. 2015 May 12;66(11):3325–37. doi: 10.1093/jxb/erv143 (PMC4449547; doi:10.1093/jxb/erv143)
Supplement: Supplementary Data [file supp_erv143_jexbot144998_file001.pdf]

Cysteine-rich receptor-like kinase CRK5 as a regulator of growth, development and ultraviolet radiation responses in Arabidopsis thaliana.

Pawel Burdiak, Anna Rusaczonek, Damian Witon, Dawid Glow, and Stanislaw Karpinski

Supplementary data

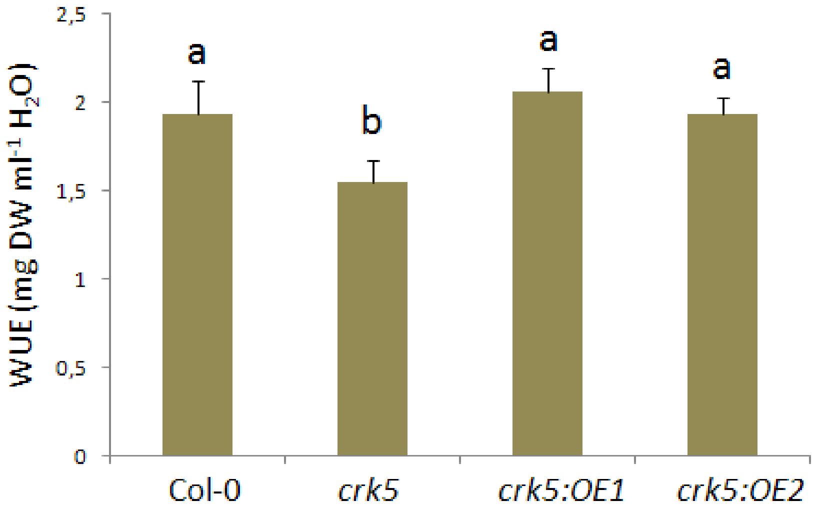

Supplementary Figure S1. Water-use efficiency (WUE) in analyzed genotypes. WUE was calculated as mg of dry weight of 5-week old plants per 1 ml of water consumed by plants. Mean values (±SD) are derived from 12 plants (n = 12). Different letters above the bars indicate a significant difference at $P < 0.005$ (Tukey's test)

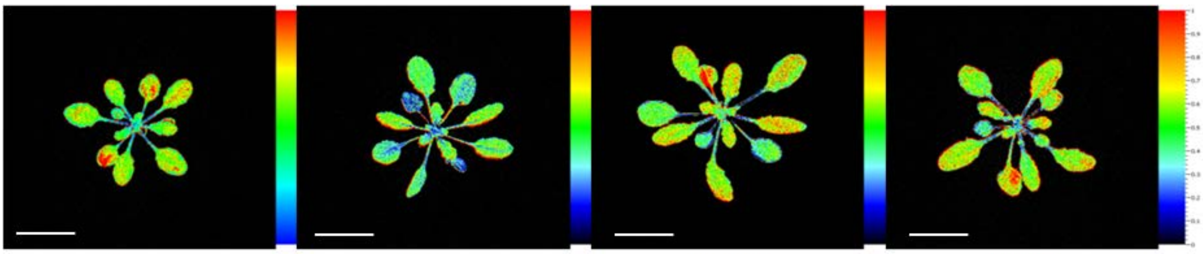

Supplementary Figure S2. Representative pictures of chlorophyll *a* fluorescence parameters. The panels display false color images of non-photochemical quenching (NPQ) of 4-week-old *Arabidopsis* rosettes. Scale bar = 2 cm.

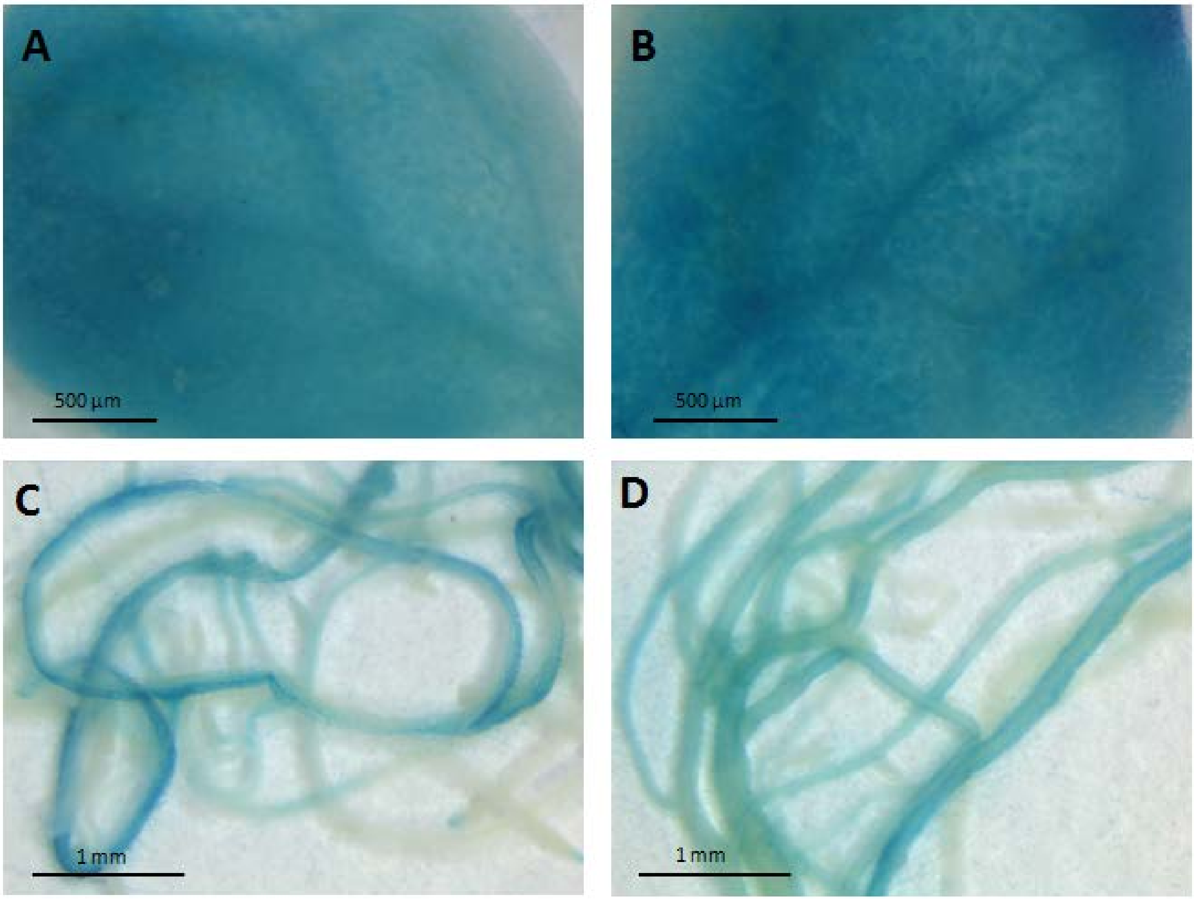

Supplementary figure S3. Histochemical GUS staining in the tissues of *CRK5 promoter::GUS* transgenic plants. The highest GUS expression was found in cotyledons (A, B) and roots (C, D).

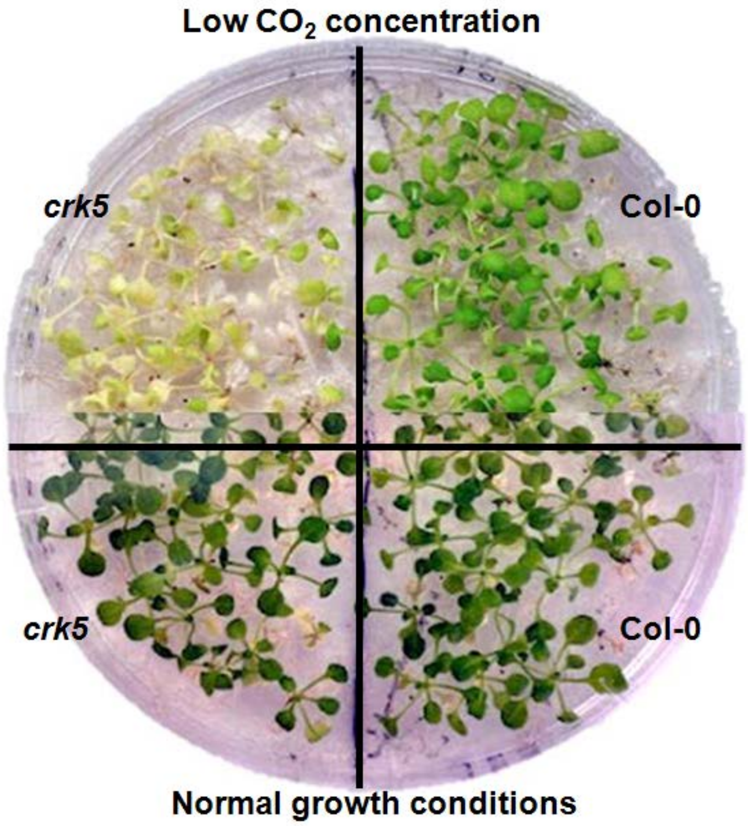

Supplementary Figure S4. Morphological phenotype of accelerated photorespiration in *crk5* after restriction of gas exchange. The plates with 2-week old seedlings were transfered to a continous light exposure (180 µM m^{-2} s^{-1}) and taped with two layers of parafilm to restrict gas exchange within Petri dishes. Photographs of separate four plates were taken before and 10 days after parafilm application and combined as a single figure.

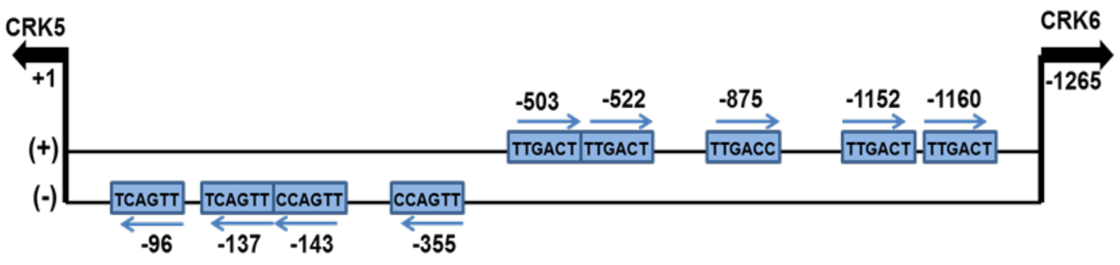

Supplementary Figure S5. Distribution of W-Box *cis*-regulatory elements in *CRK5* promoter region. These motifs are recognized and bound by WRKY transcription factors and activated *via* salicylic acid-mediated pathways.

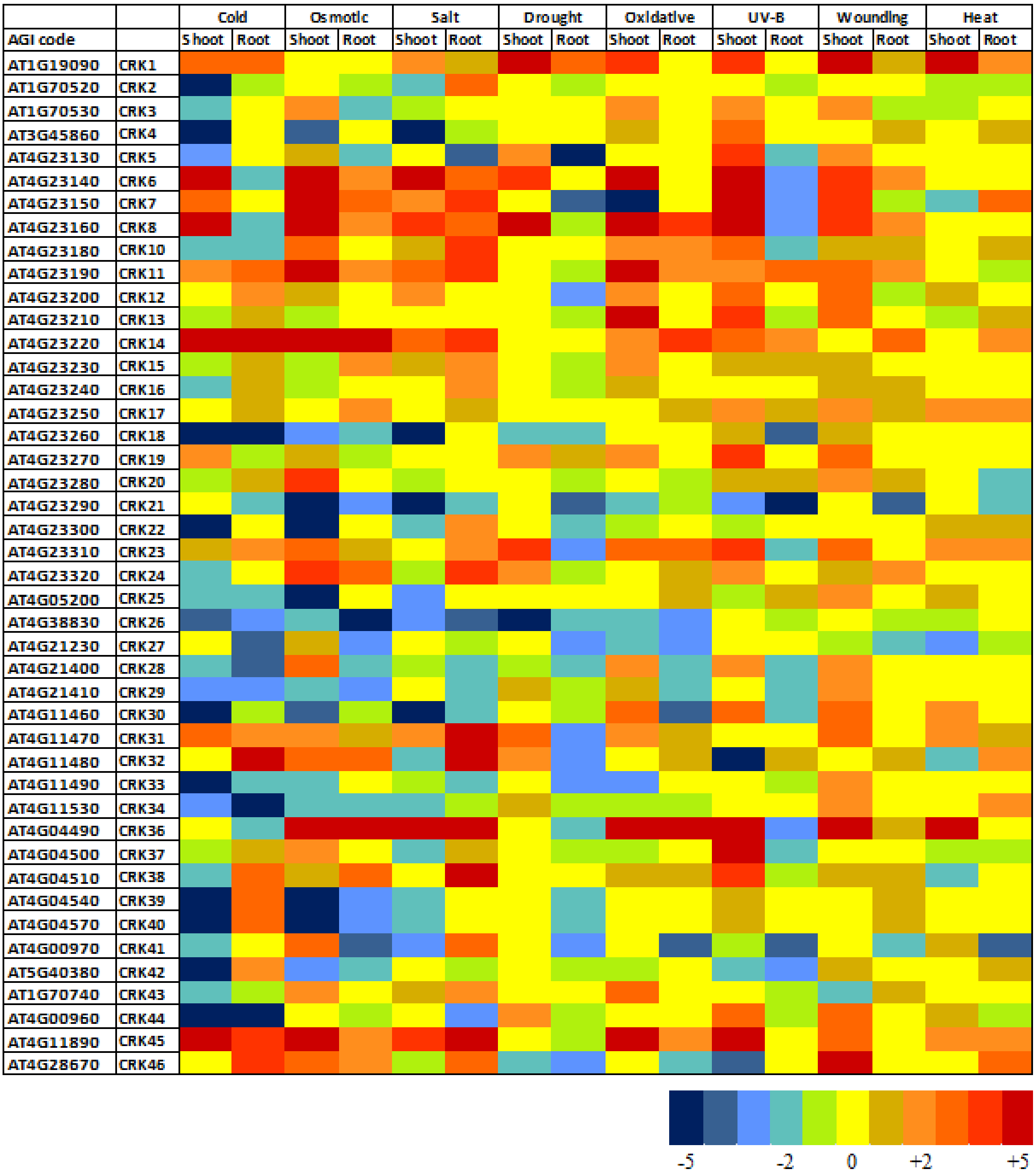

Supplementary Figure S6. Analysis of *CRKs* expression in response to abiotic stress factors. The data was collected from eFP browser (http://bar.utoronto.ca/efp/cgi-bin/efpWeb.cgi?) microarray results showing the level of CRKs transcription under different stress stimuli (cold, salt, drought, ultraviolet, wounding, heat, osmotic and oxidative stress) All results of the phenotyping were normalized and integrated into the heat-map.

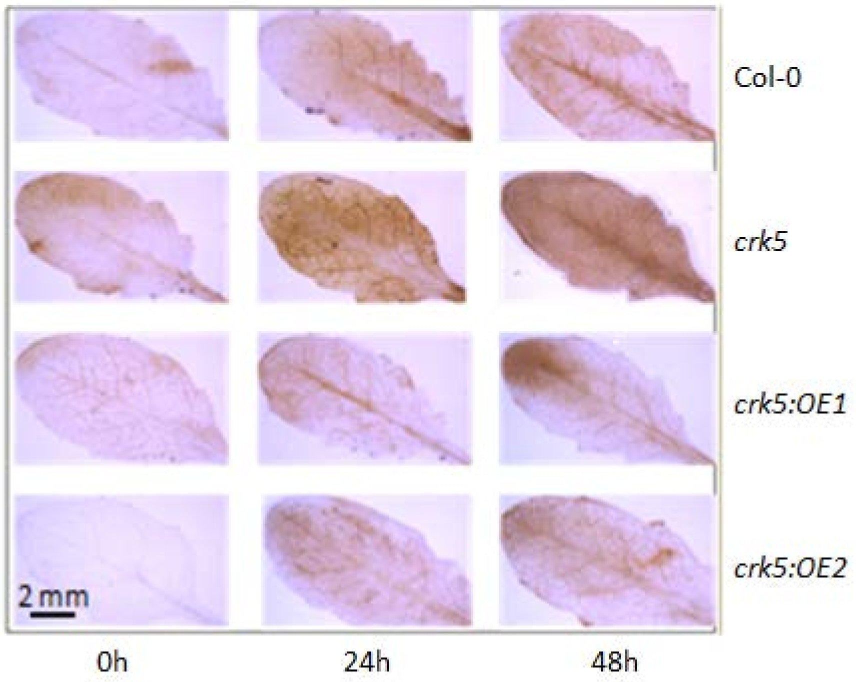

Supplementary Figure S7. Foliar content of hydrogen peroxide under growing conditions and after ultraviolet radiation episode. H_2O_2 content assessed by DAB staining.

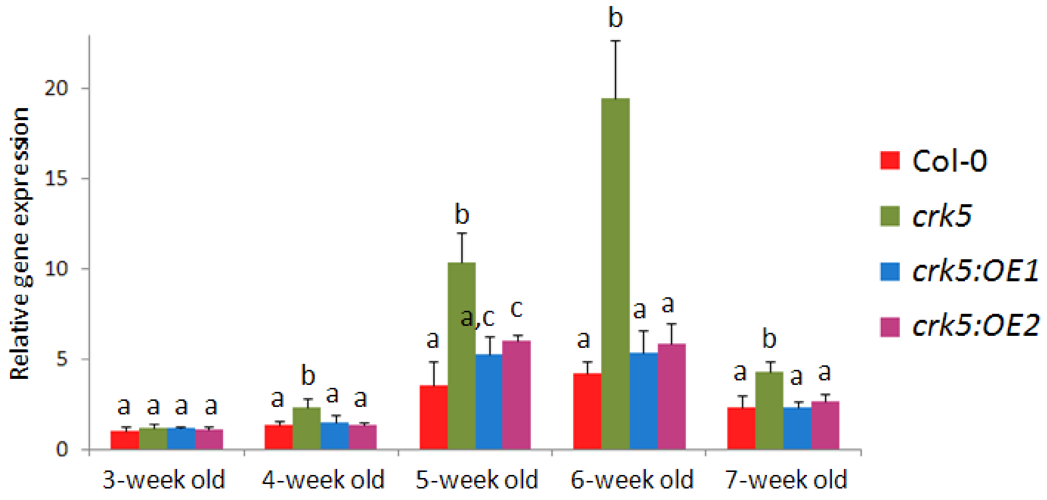

Supplementary Figure S8. Age-dependent transcript abundance for senescence marker gene *WRKY53* in analyzed genotypes. Data show relative *WRKY53* expression normalized to 3-week old wild type (Col-0) plants and represent average values ± SD. Different letters above the bars indicate a significant difference at $P < 0.005$ (Tukey's test)

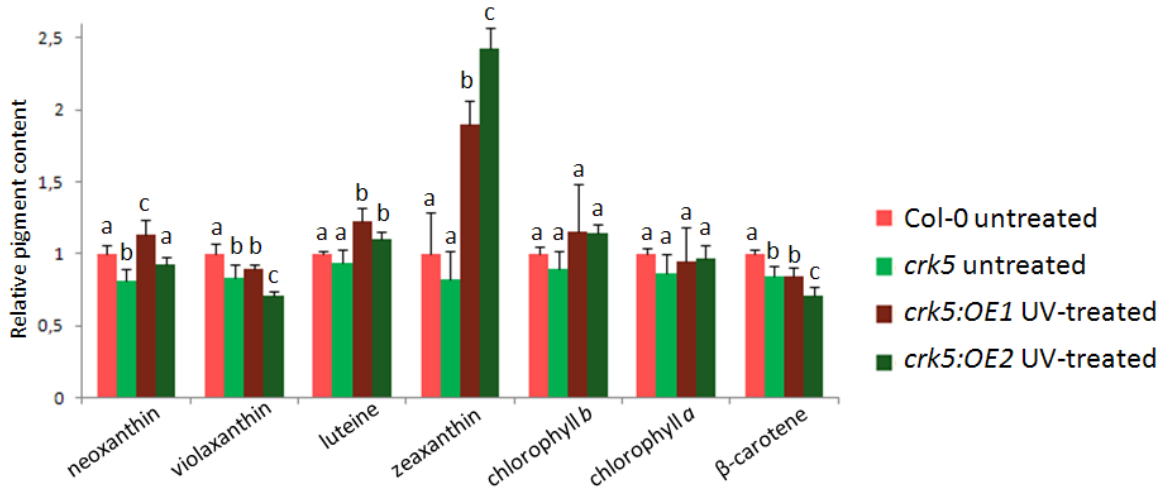

Supplementary Figure S9. Analysis of pigment content under growing conditions and 48h after UV episode. Data show relative pigment content normalized to untreated wild type (Col-0) plants and represent average values ± SD. Different letters above the bars indicate a significant difference at $P < 0.005$ (Tukey's test)

Gene name	AGI code	Sequence LP primer	Sequence RP primer	Used for
ACT2	AT3G18780	TCACCACAACAGCAGAGCGGG	GGACCTGCCTCATCATACTCGG	evaluation of RNA purity
YLS8	AT5G08290	TTACTGTTTCGGTTGTTCTCCATTT	CACTGAATCATGTTCGAAGCAAGT	qPCR reference
UPL7	AT3G53090	TTCAAATACTTGCAGCCAACCTT	CCCAAAGAGAGGTATCACAAGAGACT	qPCR reference
ERF1	AT3G23240	TTCCGATCAAATCCGTAAGC	ACCCTCTCATCGAGAAAGCA	qPCR
PDF1.2	AT5G44420	CACCCTTATCTTCGCTGCTC	GTTGCATGATCCATGTTTGG	qPCR
APX1	AT1G07890	GTCCATTCGGAACAATGAGGTTTGAC	GTGGGCACCAGATAAAGCGACAAT	qPCR
NPR1	AT1G64280	CCGCCTAAAGGAGTTTCTGA	TGTCTACAACGTCCAATAAGTGC	qPCR
PR1	AT2G14610	TACGCAGAACAACTAAGAGGCA	GGCTTCTCGTTCACATAATTCC	qPCR
WRKY53	AT4G23810	CGGAAGTCCGAGAAGTGAAG	GCCTCTCTCTGGGCTTATTC	qPCR
WRKY70	AT3G56400	TGAAGATTCCGGCGATAGTC	CTCCAAGAAAATGCGTCCTC	qPCR
CRK5	AT4G23130	TTGTTGTGCCAGTCGCTATCTCAGT	ACCCTGCAGTTGTGATGTCATCCTC	qPCR
CRK5	AT4G23130	AGGAGATCTCTCGCCAGAATC	CGATAGTCTCTTCACGGCAAC	genotyping
CRK5	AT4G23130	CACCGATTTGAGTTTCACATCATAG	TATCCAATTTCTTCACCTTTCT	amplification of *CRK5* promoter
CRK5	AT4G23130	CACCATGTCTGCTTATACCTCATTAA	ACGAGGAGCTAAAATAGTAATC	amplification of *CRK5* coding sequence

Supplementary Table S1. Primers used in this study.
